# Draft Genome Sequence of a Thermophilic Member of the *Bacillaceae*, *Anoxybacillus flavithermus* Strain Kn10, Isolated from the Kan-nawa Hot Spring in Japan

**DOI:** 10.1128/genomeA.00311-13

**Published:** 2013-05-30

**Authors:** Minenosuke Matsutani, Yasuo Shirakihara, Katsumi Imada, Toshiharu Yakushi, Kazunobu Matsushita

**Affiliations:** Department of Biological Chemistry, Faculty of Agriculture, Yamaguchi University, Yamaguchi, Japana; Structural Biology Center, National Institute of Genetics, Mishima, Shizuoka, Japanb; Department of Macromolecular Science, Graduate School of Science, Osaka University, Toyonaka, Osaka, Japanc

## Abstract

Here, we report the draft genome sequence of the *Anoxybacillus flavithermus* Kn10 strain (NBRC 109594), isolated from a water drain of the Kan-nawa Hot Spring in Japan. The draft genome sequence is composed of 90 contigs for 2,772,624 bp with 41.6% G+C content and contains 2,883 protein-coding genes and 80 tRNA genes.

## GENOME ANNOUNCEMENT

Gram-positive bacteria of the genus *Anoxybacillus* are usually found in high-temperature habitats such as geothermal springs (1). *Anoxybacillus flavithermus* is known as a major contaminant of milk powder (2). *A. flavithermus* grows in a wide range of temperatures, 30 to 72°C, and pH values, from 5.5 to 10.0 (3). The genus *Anoxybacillus* is also of interest for its biotechnological aspects, such as thermotolerant polysaccharide-degrading enzymes and even the possibility of high-temperature microbial cell factory because of the relatively small genome sizes of these organisms (4). Here, we report the draft genome sequence of a thermophilic member of the *Bacillaceae, A. flavithermus* strain Kn10, isolated from a water drain of the Kan-nawa Hot Spring at Beppu in Japan. This isolate has been deposited in the Biological Resource Center, NITE (NBRC, Kisarazu, Japan), as strain NBRC 109594. The Kn10 strain grows optimally at 55 to 60°C and does very little at 65°C.

The draft genome sequence of *A. flavothermus* Kn10 was sequenced with the next-generation sequencing platform Illumina Hiseq 2000 and generated 3,821,524 paired-end reads. The genome was assembled using the Velvet assembler ver. 1.1.02 (5), and 275× genome coverage resulted in a final assembly of 2,772,624 bp with 41.6% G+C content and an N_50_ length of 134,259 bp. Protein-coding gene prediction was performed by Glimmer 3.02 with a self-training dataset (6). tRNAs were predicted using ARAGORN (7). Functional annotation of the predicted genes was performed by BLASTP searching of the NR database (8). The draft genome sequence of *A. flavothermus* contains 90 contigs, including 52 large contigs (>1,000 bp). A total of 2,883 protein-coding genes were identified, and 80 tRNA genes were identified. Genome mapping analysis was performed by using the Bowtie 2 package with default parameters (9). Because we failed to construct the 16S rRNA sequence of Kn10 from the Illumina reads, we mapped them on the complete genome sequence of the *A. flavothermus* WK1 strain, which was isolated from the wastewater drain at the Wairakei geothermal power station in New Zealand (9, 10). The 16S rRNA sequence of Kn10 has only one nucleotide difference from that of WK1.

We found a complete set of the flagellar and chemotaxis genes and several genes for putative chemoreceptors in the draft genome sequence. It has only H^+^-coupling *motAB* (KN10_0004-0005) and no Na^+^-coupling *motPS* for the flagellar stator unit, while *Bacillus subtilis* has two *mot* genes (11). When grown at 55 to 60°C, Kn10 was highly motile, which was likely accounted for by the H^+^-coupling flagellar motility (T. Yakushi, K. Imada, and K. Matsushita, unpublished data).

### Nucleotide sequence accession numbers.

The draft genome sequence has been deposited at DDBJ/EMBL/GenBank under the accession number BARH00000000. The version described in this paper is the first version, number BARH01000000.
